# Synchronization and Bellerophon states in conformist and contrarian oscillators

**DOI:** 10.1038/srep36713

**Published:** 2016-11-09

**Authors:** Tian Qiu, Stefano Boccaletti, Ivan Bonamassa, Yong Zou, Jie Zhou, Zonghua Liu, Shuguang Guan

**Affiliations:** 1 Department of Physics, East China Normal University, Shanghai, 200241, China; 2 Institute of Condensed Matter and Material Physics, School of Physics, Peking University, Beijing, 100871, China; 3 CNR–Institute of Complex Systems, Via Madonna del Piano, 10, 50019 Sesto Fiorentino, Florence, Italy; 4 The Embassy of Italy in Tel Aviv, 25 Hamered street, 68125 Tel Aviv, Israel; 5 Department of Physics, Bar–Ilan University, 592000, Ramat Gan, Israel

## Abstract

The study of synchronization in generalized Kuramoto models has witnessed an intense boost in the last decade. Several collective states were discovered, such as partially synchronized, chimera, *π* or traveling wave states. We here consider two populations of globally coupled *conformist* and *contrarian* oscillators (with different, randomly distributed frequencies), and explore the effects of a frequency–dependent distribution of the couplings on the collective behaviour of the system. By means of linear stability analysis and mean–field theory, a series of exact solutions is extracted describing the critical points for synchronization, as well as all the emerging stationary coherent states. In particular, a novel non-stationary state, here named as *Bellerophon state*, is identified which is essentially different from all other coherent states previously reported in the Literature. A robust verification of the rigorous predictions is supported by extensive numerical simulations.

Synchronization is an emergent process of dynamical systems, wherein two (or many) interacting units adjust a given property of their motion to a collective behavior. Examples are actually ubiquitous in physics, chemistry, biology, engineering, and human society^1^,^2^. In particular, synchronization in networked oscillatory systems has attracted great attention in the past two decades, due to its many potential applications^3^,^4^. In the seventies, Kuramoto considered a paradigmatic model of globally coupled phase oscillators^5^, described by


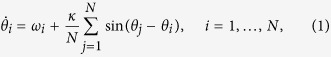


where dot denotes the temporal derivative, *θ*_*i*_ (*ω*_*i*_) is the instantaneous phase (the natural frequency chosen at random from a certain frequency distribution [FD] *g*(*ω*)) of the *i*th oscillator, and *κ* is a global coupling strength. Despite its simplicity, the model actually displays a very rich phenomenology: as the coupling strength increases, the system’s state bifurcates continuously from an incoherent regime (in which oscillators rotate according to almost their natural frequencies) into a partially coherent regime (in which part of the oscillators become phase–locked to the mean–field)^6^. Since then, the Kuramoto model and its various generalizations were taken as the standard framework for the study of synchronization of oscillatory systems, and allowed a wealth of remarkable discoveries, mostly because of the mathematical solvability and the relevance to practical applications^7^,^8^,^9^,^10^,^11^,^12^,^13^.

In the original Kuramoto model, the global coupling strength *κ* was assumed to be positive, implying that the interactions among oscillators are all attractive. However, in many cases of practical interest, the connections among units of an ensemble could be inherently suppressive, or repulsive (which would correspond, instead, to negative values of the coupling strength). For instance, both excitatory and inhibitory links are present in neural networks^14^,^15^, in cellular interactions^16^, or in social networks^17^,^18^,^19^. A first wave of studies explicitly considering attractive and repulsive interactions was inspired by models of spin glasses^20^,^21^. The coupling constant *κ* was replaced by matrix elements *κ*_*ij*_ chosen independently from a prescribed distribution, and evidence of glassy behavior^22^,^23^,^24^,^25^,^26^ was given, whose dynamical properties remain, however, still unclear^27^,^28^.

A second stream, instead, started more recently with refs 29 and 30, which generalized the Kuramoto model by replacing the coupling strength *κ* with a randomly distributed variable *κ*_*i*_, featuring either positive or negative values. The resulting model equations are


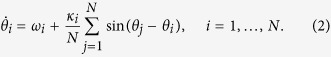


Oscillators can be then grouped into two populations: those with positive *κ*_*i*_ will behave like *conformists* (because they will attempt to follow the global rhythm of the system), whereas those with negative *κ*_*i*_ will react as *contrarians* (since they will oppose to the system’s global beat). When the system is only composed by contrarians, the emergent dynamics is fully incoherent. However, when a portion of contrarians are flipped into conformists, synchrony may appear. More precisely, when the proportion of conformists exceeds a certain threshold, the system undergoes a transition to its coherent state. Depending on the proportion of the conformists, the system exhibits both stationary states (the incoherent state, the fully coherent state, the partially synchronized *π* state with conformists and contrarians locked in anti-phase, and the traveling wave state^29^,^30^,^31^,^32^,^33^), and non-stationary (NS) states (the breathing chimera state^34^, and the Bellerophon state (including the oscillating *π* state)^35^,^36^). Here, the stationary state refers to such an asymptotic state of the dynamical system in which the probability density function is time-independent in certain rotating frame, and non-stationary state otherwise.

This Manuscript provides a full analytical treatment of model (2), under the assumptions that the coupling strengths are chosen from a binary set (*κ*_*i*_ ∈ {*κ*_−_, *κ*_+_}, with *κ*_−_ < 0 for contrarians and *κ*_+_ > 0 for conformists), and that the FD is a symmetric Cauchy–Lorenz probability density *g*(*ω*) = *γ*/[*π*(*ω*^2^ + *γ*^2^)] of width *γ* and vanishing median. We consider then three distinct strategies to flip contrarians into conformists. (1) Contrarians are randomly chosen, in a first case, and flipped into conformists. (2) Contrarians are ranked, in a second case, according to the absolute value of their natural frequencies |*ω*_*i*_|, and then orderly flipped into conformists from the largest |*ω*_*i*_| to the smallest (i.e. the coupling strength of the *i*th oscillators is *κ*_*i*_ = *κ*_+_ if |*ω*_*i*_| > *ω*_0_, and *κ*_*i*_ = *κ*_*−*_ otherwise, being *ω*_0_ ≥ 0 a specific parameter). Let *p* denotes the proportion of conformists in the system, then 
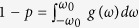
. (3) Contrarians are ranked, in the third case, with a strategy which is the inverse of that of the second case (i.e., now *κ*_*i*_ = *κ*_+_ if |*ω*_*i*_| < *ω*_0_, and *κ*_*i*_ = *κ*_−_ otherwise). Here 
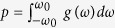
. The three strategies correspond, in fact, to three different correlations between the distribution of the coupling strengths and the natural frequencies, respectively given by


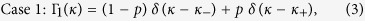



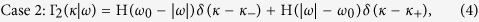



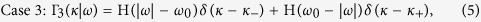


where *p* ∈ [0, 1] is the fraction of conformists in the system, and H(·), *δ*(·) are the Heaviside and Dirac delta distributions, respectively. In this work, *p*, which depends on *ω*_0_, is used as the control parameter for studying the system’s transitions. Given a value of *p*, the proportion of conformists and contrarians in the system are fixed, and then the evolution of Eq. (2) is investigated. While Case 1 coincides with the Hong–Strogatz model^29^, and Case 2 and Case 3 were only numerically investigated in ref. 35, we here offer a unified analytical treatment encompassing all the cases. In particular, by linear stability analysis and mean–field theory arguments, we give the exact solution of Eq. (2) for the critical proportions of conformists needed to attain synchrony, and we identify all the possible coherent states (and all the bifurcations among them). Further, we report the existence of a novel non–stationary coherent state in model (2), characterized by quantized coherent clusters of oscillators, whose phases are neither phase–locked, nor frequency–locked. Due to their specular resemblance with chimera states^37^,^38^,^39^, the new states have been termed *Bellerophon states* (BS)^36^, as Bellerophon was the hero who, in the Greek mythology, confronted with (and eventually killed) the monster Chimera. All the theoretical results are then robustly verified by extensive numerical simulations.

## Results

### Linear stability analysis

In the mean–field form, Eq. (2) can be rewritten as


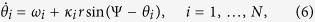


where *r* and Ψ define the complex order parameter


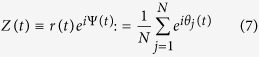


quantifying the instantaneous collective rhythm produced by the ensemble.

In the thermodynamic limit (*N* → ∞) one can define an oscillator density *ρ*(*θ*, *t*|*ω*, *κ*) on the (*θ*, *t*)–space, which gives the probability to find an oscillator with phase *θ*, frequency *ω* and coupling *κ*_*i*_ at time *t*. *ρ* satisfies the normalization condition 
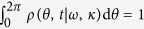
 for each *ω*, *κ*_*i*_ and *t*, and its evolution is ruled by the continuity equation ∂_*t*_*ρ* + ∂_*θ*_(*υ*_*θ*_*ρ*) = 0, being


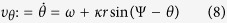


the velocity field on the circle. With the above definitions, the order parameter (7) can now be casted in the form 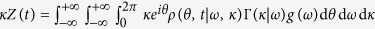, and the continuity equation satisfied by *ρ* changes accordingly (see Eq. (22) in the Methods). It is easy to check that *ρ*_0_(*θ*, *t*|*ω*, *κ*) = (2*π*)^−1^ is the stationary solution of the latter equation corresponding to the desynchronized phase, i.e. the regime where *Z* ~ 0. To predict the critical points where the incoherent phase loses stability, one linearly perturbs the related density, so that 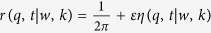, where *ε* ≪ 1 and *η*(*θ*, *t*|*ω*, *κ*) is a perturbation function that can be expanded as a Fourier series in *θ*. Since only the first harmonic *c*(*ω*, *κ*, *t*) contributes nontrivially to the dynamics of the linearized amplitude equation (see the Methods for further details), one can safely write the perturbation function in the form *η*(*θ*, *t*|*ω*, *κ*) = *c*(*ω*, *κ*, *t*)*e*^*iθ*^. Inserting the latter into the continuity equation, one arrives at a linearized characteristic equation of the form 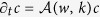, where 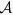 is a linear integral operator defined as


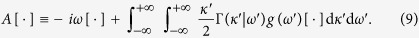


The linear stability properties of the incoherent phase depends then on the spectral properties of operator (9).

The spectrum of 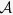 contains both continuous and discrete sets. Following the analysis carried out in ref. 7, the continuous part of the spectrum of 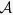 is purely imaginary, {−*iω* : *ω* ∈ Support(*g*)}, which is hence the whole imaginary axis for a Cauchy–Lorenz FD. This implies that the incoherent state in model (2) can never be linearly stable, being either unstable or neutrally stable. As for the discrete part of the spectrum of 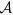, one instead seeks for solutions of the form *c*(*ω*, *κ*, *t*) = *b*(*ω*, *κ*)*e*^*λt*^, so that the characteristic equation takes now the form (see again the Methods for details)


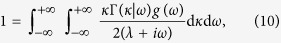


which holds for every 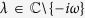. Equation (10) implicitly relates the proportion of conformists *p* ∈ [0, 1] (or, equivalently, the control parameter 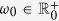) with the discrete eigenvalue *λ*, allowing one to predict the critical fraction *p*^*c*^ for the forward phase transition (PT) at which the incoherent state loses its stability. We stress that both real and imaginary parts of *λ* ≡ *x* + *iy* (with 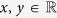) are affecting the incoherent state’s stability, so that Eq. (10) has to be in general splitted into a system of two coupled equations (see Eqs (28 and 29) in the Methods). For the sake of simplicity, we here concentrate on the discussion of the three distinct cases mentioned above, while addressing the reader to the Methods for all details on the calculations for the critical thresholds.

Case 1. Inserting the expression (3) for the distribution of strengths into Eq. (10), yields


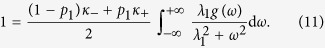


Since the left–hand side of the latter equation is real, any eigenvalue *λ*_1_ must satisfy the condition *y*_1_ = 0. However, in contrast with the classical Kuramoto model^8^, *x*_1_ is not necessarily positive as *κ*_−_ < 0. Therefore, by increasing *p*_1_, the incoherent state will eventually lose its stability if *x*_1_ changes from negative to positive. Imposing the critical condition *x*_1_ → 0 for Eq. (11), one has that the critical proportion of conformists for the forward PT is


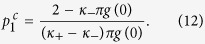


In particular, for a Cauchy–Lorenz FD, one obtains


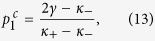


which is consistent with Eq. (12) in ref. 29.

**Figure 1:**
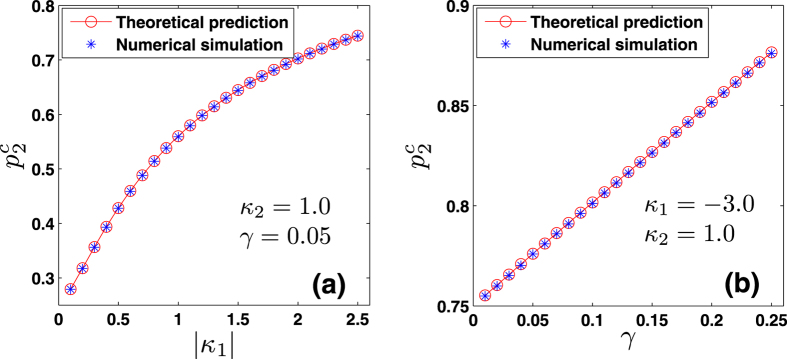
Corroboration of the analytical predictions for the forward transition’s critical point in Case 2. (**a**) Monotonic behavior of 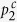 *vs.* |*κ*_1_|, for *κ*_2_ = 1.0 and *γ* = 0.05. (**b**) 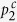 *vs. γ*, for *κ*_1_ = −3.0 and *κ*_2_ = 1.0. 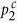 increases almost linearly, as *γ* increases. Numerical integrations of Eq. (2) are performed by a fourth-order Runge-Kutta method with time step 0.01, *N* = 50,000, and random initial conditions for the phase variables.

Case 2. Substituting formula (4) into Eq. (10) (and applying the critical condition *x*_2_ → 0) yields eventually (see the Methods for further details) to the following implicit expression for 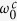:


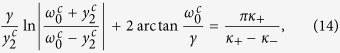


where the critical values of 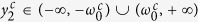 are


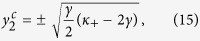


for a Cauchy–Lorenz FD. Note that Eqs (14 and 15) are consistent with Eq. (12) in ref. 35. In particular, Eq. (15) implies that 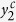 exists only if *κ*_+_ > 2*γ*, suggesting that *x*_2_ → 0 is self–contradictory when *κ*_+_ < 2*γ*. Therefore, the real part of *λ*_2_ must be negative when *κ*_+_ < 2*γ*, being the positive case physically unreasonable. A conclusion is that the coherent state will never emerge if *κ*_+_ < 2*γ* (no matter how large the population of conformists is), as observed by numerical simulations in ref. 35. Then, a forward PT can only occur if *κ*_+_ > 2*γ*, in which case Eq. (14) gives us an implicit formula for the critical proportion 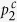 of conformist, namely


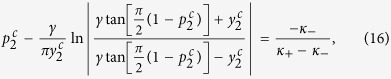


being 
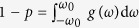
. Solving numerically Eqs (15 and 16), one obtains the behavior of 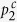 in terms of the parameters *κ*_1_, *κ*_2_ and *γ*, as shown in Fig. 1. It is really remarkable the way such an analytical prediction is endorsed and validated by direct numerical integrations of Eq. (2).

**Figure 2:**
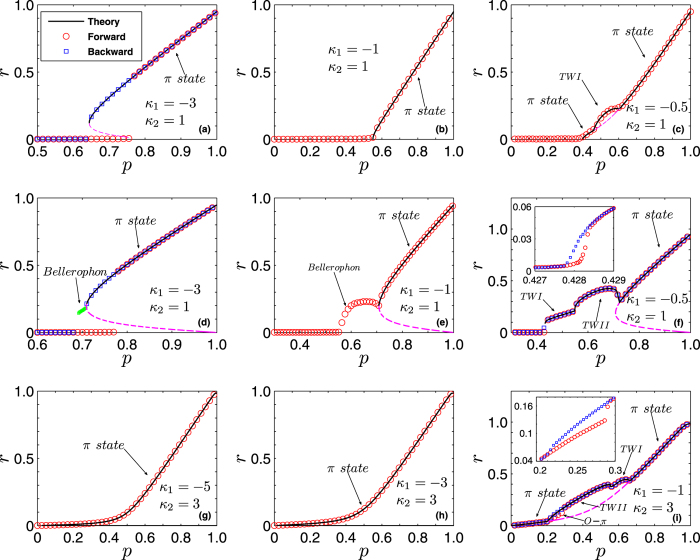
Synchronization in Eq. (2) as the proportion of conformists increases. From top to bottom, the three rows correspond to Case 1, Case 2 and Case 3, respectively. From left to right, the three columns correspond to the case of |*κ*_−_| > *κ*_+_, |*κ*_−_| = *κ*_+_, and |*κ*_−_| < *κ*_+_, respectively. Both the forward (red circles) and the backward (blue squares) transitions are studied in an adiabatic way, and the black (pinkish red) curves correspond to theoretical predictions of the stable (unstable) stationary coherent states, including the *π* state, the TW–I state, and the TW–II states. The O–*π* state denotes the oscillating *π* state that is non-stationary^35^. All the above results are obtained for a Cauchy–Lorenz FD with *γ* = 0.05. Other stipulations as in the Caption of Fig. 1.

**Figure 3:**
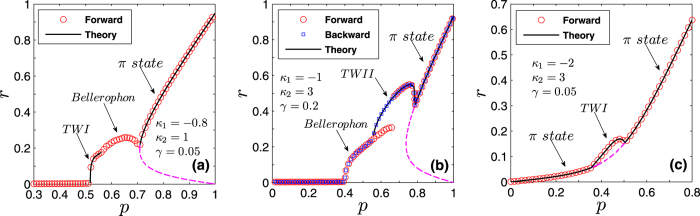
Further characterization of the solution of Eq. (2) as the proportion of conformists increases. (**a**,**b**) Refer to Case 2, while (**c**) refers to Case 3. Here *γ* = 0.05, 0.2, 0.05 for (**a**–**c**), respectively. All stipulations are the same as in the caption of Fig. 2.

Case 3. Inserting the distribution (5) into Eq. (10), and following analogous reasoning as in the previous case (see the Methods for details), one can conclude that the forward PT never occurs when *κ*_+_ < 2*γ*, while the following implicit relation for 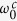 holds (as *x*_3_ → 0) for *κ*_+_ > 2*γ*:


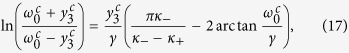


where the critical values of 
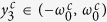
 are given again by Eq. (15). Notice that 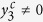 as far as *κ*_+_ > 2*γ*, and it can be shown (see the Methods for details) that Eq. (17) has no solution as a function of 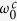. This suggests that, when *κ*_+_ > 2*γ*, the condition *x*_3_ → 0 is self–contradictory, so that the real part of *λ*_3_ must be either positive or negative. A negative value of *x*_3_ means that the incoherent state is always neutrally stable, no matter how large the coupling strength is, which is physically unreasonable. A positive value of *x*_3_, instead, implies that the critical proportion of conformist vanishes (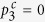) as the incoherent phase will lose stability. The zero threshold for synchronization in Case 3 can be heuristically understood as follows. From Eq. (6), one can see that the phase–locking condition for conformists is |*ω*_*i*_| ≤ |*κ*_*i*_*r*|. Thus the smaller the absolute value of the natural frequency is, the easier for the conformists is to get synchronized. In Case 3, contrarians are flipped into conformists from the smallest |*ω*_*i*_|. Therefore, the forward PT will take place as soon as *p*_3_ deviates from zero. Once again, numerical simulations fully confirm the existence of the zero threshold in this case, as shown in Figs 2 and 3(c).

### Mean–field theory for stationary states

While linear stability allows unveiling the critical thresholds for the forward PTs of model (2) in all the three cases, for a better description of the scenario emerging after the incoherent state becomes unstable one actually needs to solve the continuity equation related to the model. This task constitutes a mathematical challenge [even for the classical Kuramoto model (1)] and has inspired the development of several elegant and ingenious techniques^12^. In what follows, we will adopt the self–consistent arguments introduced by Kuramoto^5^,^6^, in order to predict all the possible *stationary* states of system (2), as the proportion of conformist increases.

For stationary coherent states, the amplitude *r* defined in Eq. (7) is constant, and the mean–field phase Ψ rotates uniformly with frequency Ω, i.e. Ψ(*t*) = Ω*t* + Ψ_0_ (without loss of generality, one can further set Ψ_0_ = 0, after an appropriate time shift). Moving into the rotating frame with frequency Ω, one can set *ϕ*_*i*_ ≡ *θ*_*i*_ − Ψ, so that the mean–field Eq. (6) has now the form


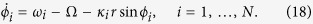


Notice that, due to the asymmetry in the coupling parameters *κ*_*i*_, Ω = 0 is not in general warranted. Eq. (18) exhibits two types of long–terms behavior^8^. When |*ω*_*i*_ − Ω| ≤ |*κ*_*i*_*r*|, Eq. (18) approaches a stable fixed point defined by the expression 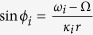, corresponding to phase–locked oscillators entrained by the mean–field. When |*ω*_*i*_ − Ω| > |*κ*_*i*_*r*|, oscillators are instead drifting around the circle, running in a non–uniform manner. As both populations of oscillators contribute to the stationary order parameter (7), one can write 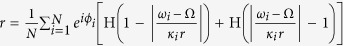.

In the thermodynamical limit, the sums in the above expression are replaced by integrals over the space of probability distributions. Therefore, taking trace of the contributions coming from the phase–locked and the drifting oscillators, and equating real and imaginary parts of the above expression (see the Methods for further details), one obtains


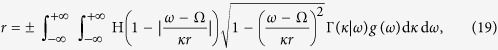



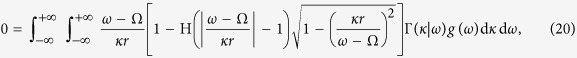


where conformists and contrarians correspond, respectively, to the positive and negative sign before the integral in Eq. (19). Taken together, Eqs (19 and 20) provide a closed system of self–consistent equations for the dependence of the amplitude *r* and frequency Ω of the mean–field on the system’s parameters.

As a general property of Eqs (19 and 20), it is worth noticing that Ω = 0 is always a trivial solution of the phase balance Eq. (20), corresponding to the so–called *π*–state reported in ref. 29. In such a state, conformists and contrarians organize collectively into a partially synchronized behavior where they both satisfy a stationary distribution of phases, and the phase difference between the two clusters is always *δ* = *π*. States with Ω ≠ 0 can also be solutions of Eq. (20), and they correspond to travelling–wave (TW) states, where the two clusters of contrarians and conformist always maintain a constant phase–separation *δ* ≠ *π*, while rotating with the same frequency along the unit circle. We here report on the existence of *two types* of TWs in model (2): in the first type (hereafter referred to as TW–I, and already observed in refs 31, 35, 40 and 41), the coherent oscillators form a giant connected cluster in terms of the instantaneous frequencies; by contrast, in the second type (TW–II) the coherent oscillators form two giant clusters, separated by a sea of drifting contrarians.

With all this in mind, we are now in the condition of identifying all possible stationary coherent states of model (2) for the three considered flipping strategies. To keep the formalism at a minimum, we here focus on the full characterization of such states (and the amazingly good confirmations of the predictions by numerical simulations), while addressing the reader to the Methods for all relevant details.

Case 1. Inserting expression (3) for Γ_1_(*κ*) into Eqs (19 and 20), one obtains a system of two self–consistent equations for the order parameters *r*_1_ and Ω_1_ (Eqs (45 and 46) in the Methods, respectively).

For those stationary states for which Ω_1_ = 0, the system simplifies to


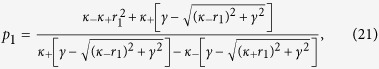


which is consistent with Eq. (13) in ref. 29. From such an expression, one can extract the critical proportion of conformists for both the forward and backward PTs. Performing a numerical stability analysis, one observes that when |*κ*_−_| ≤ |*κ*_+_|, the system undergoes a supercritical bifurcation where a stable *π* state emerges from the incoherent state, while for |*κ*_−_| > |*κ*_+_|, the system undergoes a subcritical bifurcation and an unstable *π* state emerges, together with hysteresis^29^. When *p*_1_ = 1, we get 
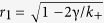
, which is consistent with the results of section 4 in ref. 8.

When Ω_1_ ≠ 0, numerical solutions of Eqs (45 and 46) of the Methods allow to identify the TW–I state. Figure 2(a–c) report the theoretical predictions, and show the bifurcations among the various phases of the system. In particular, one finds that, when |*κ*_−_| > |*κ*_+_| [Fig. 2(a)], the incoherent state loses its stability via a subcritical bifurcation as the population of conformists *p*_1_ is increased. Since the local *π* state is unstable near the bifurcation point 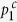, the system suddenly jumps onto another stable *π* state, through a discontinuous (forward) PT. For the backward PT (as *p*_1_ is decreased), the stable and unstable *π* states gradually approach each other and eventually collide at the bifurcation point 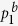 (see the Methods for details). The transition is discontinuous, and it occurs at a critical point 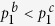, showing hence hysteresis. When |*κ*_−_| = |*κ*_+_| [Fig. 2(b)], the transition between the incoherent state and the stable *π* state is continuous. When |*κ*_−_| < |*κ*_+_| [Fig. 2(c)], the system progressively experiences (as *p*_1_ increases) several continuous PTs going through the incoherent state, the *π* states, the TW–I state, and finally again to the *π* state. All the theoretical predictions are amazingly well supported by extensive numerical simulations, and are fully consistent with the results of ref. 29. Further numerical results show that the mean–field solutions of TW–I state are always unstable when |*κ*_−_| > |*κ*_+_|. At variance, they can be stable when |*κ*_−_| < |*κ*_+_|, only in an intermediate range of *p*_1_ when *γ* is sufficiently small.

Case 2. Substituting Eq. (4) into Eqs (19 and 20), one obtains a system of two self–consistent equations for *r*_2_ and Ω_2_ (Eqs (49 and 50) in the Methods, respectively).

One can identify the entire *π* state, and predict the critical proportions of conformists where the *π* state loses its stability in the forward and backward PTs (see the Methods for further details). When *p*_2_ = 1, i.e., *ω*_0_ = 0, we get 
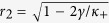
 from Eq. (49), which is consistent with the results of ref. 8 again. Analogously, by performing a numerical stability analysis of those states for which Ω_2_ ≠ 0, one can predict all the possible TW states, and characterize the corresponding forward and backward PTs. Figure 2(d–f) report all these theoretical predictions, together with direct numerical simulations of Eq. (2). When |*κ*_−_| > |*κ*_+_|, Fig. 2(d) shows that the forward transition is discontinuous, and the incoherent state of the system jumps onto the *π* state. For the backward transition, instead, the stable and unstable *π* states initially approach and collide. Remarkably, the system does not directly return to the incoherent state after the collision, but it bifurcates into a non–stationary state (the so–called *Bellerophon state*, see details in the next section) through a continuous transition. Then, as *p*_2_ further decreases, the system transits into the incoherent state discontinuously. When |*κ*_−_| = |*κ*_+_| and as *p*_2_ increases [Fig. 2(e)], the system first bifurcates with continuous PTs into the Bellerophon state, and then to the *π* state. When |*κ*_−_| < |*κ*_+_| [Fig. 2(f)], the system successively bifurcates through continuous transitions into the TW–I state, the TW–II state, and finally the *π* state. For some parameters, the successive bifurcations could be the TW–I state, the Bellerophon state, and finally the *π* state, as shown in Fig. 3(a). Furthermore, it is also found that as *p*_2_ increases, the Bellerophon state may occur even before the TW–II state, as shown in Fig. 3(b) where *γ* = 0.2.

Case 3. In analogy with Case 2, the insertion of Eq. (5) into Eqs (19 and 20) yields a closed system of self–consistent equations for the order parameters *r*_3_ and Ω_3_ (see Eqs (54 and 55) in the Methods).

Also in this case one can characterize the *π* state, and the critical proportion of conformists for the forward and backward PTs (see the Methods for further details). When *p*_3_ = 1, i.e., *ω*_0_ → +∞, we get 
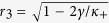
 from Eq. (54), which is supported by the results of ref. 8. Furthermore, one can obtain a complete characterization of the TW states and the associated bifurcations. Figure 2(g–i) report the theoretical predictions, and once again the remarkably good verification given by direct numerical simulations of Eq. (2). For both |*κ*_−_| > |*κ*_+_| [Fig. 2(g)] and |*κ*_1_| = |*κ*_2_| [Fig. 2(h)], the *π* state is stable, and the critical proportion of conformists vanishes (i.e. 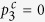, as predicted by the linear stability theory). When |*κ*_1_| < |*κ*_2_| and as *p*_3_ increases [Fig. 2(i)], the system sequentially bifurcates into a *π* state, a non–stationary state (known as the *oscillating π* [O–*π*] state^35^), a TW–II state, a TW–I state, and eventually a *π* state. All involved transitions are continuous except the first one (for which, instead, the forward transition is discontinuous), as shown in the inset of Fig. 2(i). Notice that, for some other parameters, a different sequence of bifurcations (made of a *π* state, followed by a TW–I state, and eventually again a *π* state) emerges, as reported in Fig. 3(c).

### The Bellerophon state

A novel coherent state (unnoticed in ref. 35 and here called the Bellerophon state) is discovered in model (2). Such a state occurs in Case 2, when |*κ*_1_| ≥ |*κ*_2_| [see Fig. 2(d,e)], and it is intrinsically different from any other previously observed coherent states in Kuramoto-like models. It is, indeed, a non-stationary state, with multiple coherent clusters.

**Figure 4:**
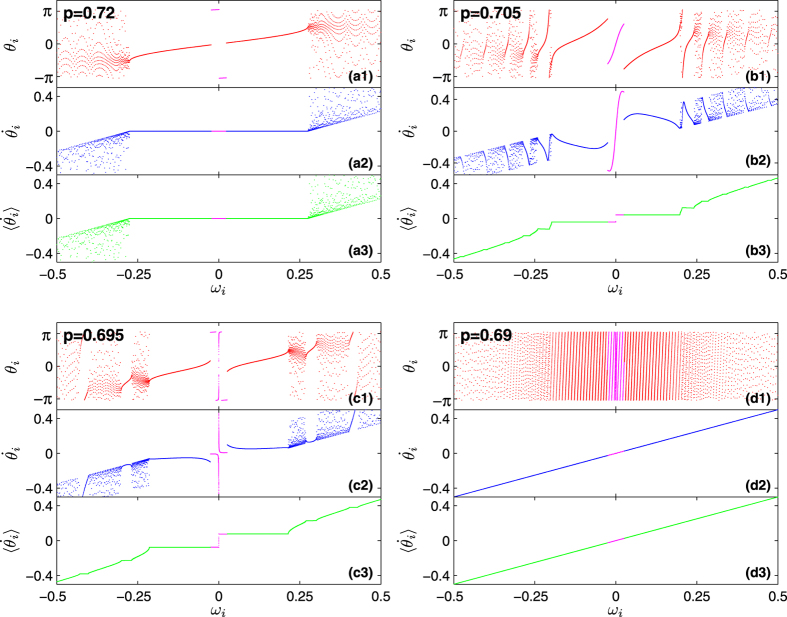
Typical states emerging in Case 2 along the backward transition of Fig. 2(d). Snapshots of the instantaneous phase *θ*_*i*_ (upper plots), the instantaneous frequency (speed) 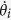 (middle plots), and the average frequency (average speed) 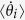 (lower plots) *vs.* natural frequencies {*ω*_*i*_} of the oscillators. (**a**) The *π* state with *p* = 0.72. (**b**,**c**) The Bellerophon states with *p* = 0.705 and *p* = 0.695, respectively. (**d**) The incoherent state with *p* = 0.69. The pink color is used to mark contrarian oscillators. All other color are used for conformists.

Figure 4 illustrates four typical states in the backward transition corresponding to Fig. 2(d), in terms of the instantaneous phases *θ*_*i*_, the instantaneous frequencies (or speeds) 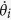, and the average frequencies (average speeds) 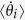 of the oscillators. When *p* = 0.72 [Fig. 4(a)], the system is in the *π* state. When *p* = 0.69 [Fig. 4(d)], the system has completed the backward transition and is in the incoherent state. For intermediate values of *p* (0.69 < *p* < 0.71), the Bellerophon state emerges. For instance, Fig. 4(b,c) report the cases of *p* = 0.705 and *p* = 0.695, respectively. There, it is easy to see that oscillators split into multiple coherent clusters: two clusters of contrarians, and many pairs of clusters of conformists.

The most important feature of the Bellerophon state is that, within each coherent cluster, neither the phases nor the instantaneous frequencies of the oscillators are locked. They actually correlate with each other in certain ways [see the cusped patterns in Fig. 4(b2) and (c2)] so that the average frequencies lock to certain constants [see the staircases structure in Fig. 4(b3) and (c3)]. It should be noticed that similar cusped pattern characterizes the average speeds of the oscillators in chimera states.

**Figure 5:**
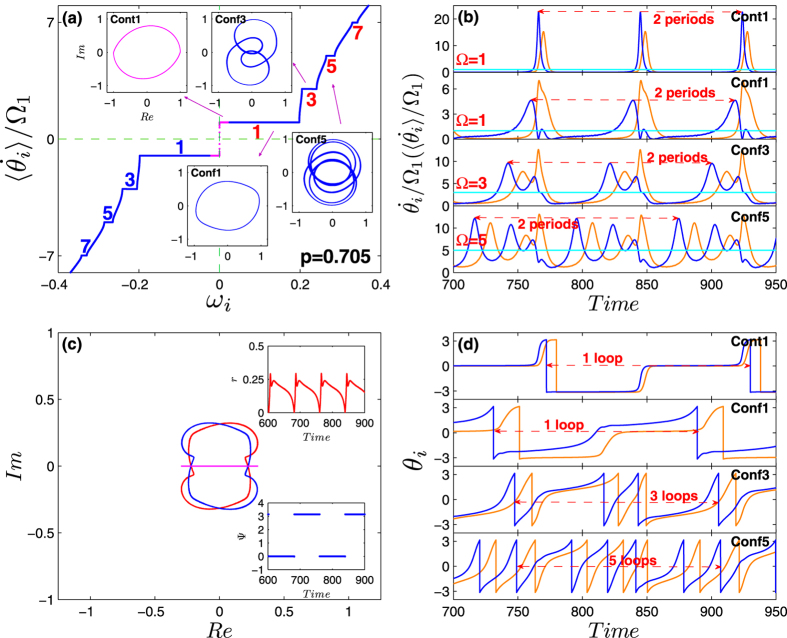
Characterization of the Bellerophon state of Fig. 4(b). (**a**) The staircases of the average speeds for coherent clusters, corresponding to the odd-numbered multiples of the fundamental frequency Ω_1_. The insets plot the local order parameter (i.e., that contributed by only those oscillators in a certain cluster) in the complex plane, for clusters *Cont*1, *Conf*1, *Conf*3 and *Conf*5 (*n* = 1, 3, 5), respectively. Note that the average frequencies of clusters *Cont*1, *Conf*1 are the same. (**b**) Time series of the instantaneous speeds of clustered oscillators. In each panel, two sample oscillators are arbitrarily chosen from clusters *Cont*1 (top), *Conf*1 (second), *Conf*3 (third) and *Conf*5 (bottom). Straight lines mark the average speed. The instantaneous speeds of oscillators inside the same cluster evolve periodically, but different oscillators follow different periodic patterns. However, the average speeds during one period for all oscillators in a certain cluster are the same. Interestingly, it is found that the speeds of contrarians in the coherent cluster turn out to be intermittent. They almost statically rest for most of time, and then burst (rotating) for a while. Note that in (**a**,**b**), the vertical axis has been normalized by the fundamental frequency Ω_1_. (**c**) The order parameters for all oscillators (including the drifting ones) with positive (blue oval) and negative (red oval) frequencies, and the order parameter for all oscillators (the pinkish red line). The insets are the time series of the global order parameters *r*(*t*) and Ψ(*t*), which are typically oscillatory. (**d**) Time series of the instantaneous phases corresponding to (**b**). During one period *T*1 = 1/Ω_1_, the oscillators in *Cont*1 and *Conf*1 all perform one loop along the unit circle, and in the mean time, the oscillators in *Conf*3 and *Conf*5 rotate three loops and five loops, respectively. Compared with (**b**), it is found that the speeds of all coherent oscillators actually experience two periods within the time *T*1.

In order to gather a better insight on Bellerophon states, we focus on the specific example of Fig. 4(b), and we further characterize in Fig. 5 the quantitative aspects of it. As shown in Fig. 5(a), the staircase structure of oscillators’ average frequencies distributes symmetrically with respect to natural frequencies. In between two adjacent clusters there are drifting oscillators (both contrarians and conformists). For clustered oscillators, the structure of average frecuencies includes a fundamental (lowest) frequency Ω_1_, and a series of other (higher) values that are all odd multiple of it^42^, i.e., Ω_±*n*_ = ±(2*n* − 1)Ω_1_ with *n* = 1, 2, 3, …. Therefore the gap between two neighboring clusters in the staircase is twice the fundamental frequency Ω_1_. In the Bellerophon state, there are multiple clusters of conformists, which can be denoted by *Conf* [±(2*n* − 1)] with *n* = 1, 2, 3, …. For the contrarians, there are only two synchronous clusters around *ω* = 0, which can be denoted as *Cont*(±1). This is just because in Case 2 contrarians are flipped into conformists following the inverse order of the natural frequencies.

Figure 5(b) shows that the instantaneous frequencies of oscillators inside the same cluster evolve periodically, but different oscillators follow different periodic patterns. In other words, the instantaneous speed for each oscillator evolves *uniquely*. This makes Bellerophon states essentially different from all other coherent states observed in Kuramoto-like models, in which oscillators are typically frequency-locked inside the coherent cluster. However, the average frequencies during one period for all oscillators in a certain cluster turn out to be the same, i.e., an odd-numbered multiple of Ω_1_. As the instantaneous frequency characterizes the rotations of oscillators along the unit circle, very interesting collective motion of oscillators is observed [see Fig. 5(d)]: during one period *T*_1_ = 1/Ω_1_, the oscillators in *Cont*(±1) and *Conf*(±1) all perform one loop along the unit circle, and in the mean time, the oscillators in *Conf*(±3) and *Conf*(±5) rotate three loops and five loops, respectively. In analogy, oscillators in *Conf *[±(2*n* − 1)] will perform (2*n* − 1) loops. In camparison with Fig. 5(b), one further finds that during one period *T*_1_, the instantaneous frequency for all coherent oscillators experiences two periods, i.e., each oscillator repeats its speed during the two half periods. Since the oscillators inside one coherent cluster are not frequency-locked, the order parameters depict complicated orbits, as shown in the insets of Fig. 5(a). As a consequence, the resulting order parameter exhibits an oscillatory pattern, and its phase turns out to be binary as shown in Fig. 5(c).

Furthermore, the motion of oscillators turn out to be intermittent within the Bellerophon state, especially for those oscillators corresponding to small natural frequencies, such as clusters *Cont*(±1) and *Conf*(±1). As shown in Fig. 5(b,d), the dynamics of these clusters exhibits two distinct stages, i.e., the bursting stage and the resting stage. The former corresponds to a fast rotation, while in the latter oscillators are almost static. For example, clusters *Cont*(±1) and *Conf*(±1) are at the resting stage for most of time and at the bursting stage only for a short period. In our simulations, it is found that clusters *Cont*(±1) and *Conf*(±1) always exhibit the strongest intermittency, which makes them behave like the *π* state during most the time. In addition, it is also revealed that as control parameter *p* decreases, the intermittency becomes weaker, implying that the system gradually moves away from the *π* state. For an even better visualization of all dynamical features of the Bellerophon state (including the intermittency-like behavior characterizing the motion of the instantaneous phases within each cluster), two animated movies are enclosed in the Supplementary Information (SI) which directly report the time evolution of phases, speeds, and rotations of oscillators on the unit circle.

Based on above analysis, the Bellerophon state is understood as a weaker form of coherence achieved by the coupled oscillators when the control parameter is at an intermediate value. In other words, like the TW state, it can be regarded as a transitional state between the incoherent state (full asynchrony) and the *π* state (full synchrony): on the one hand the control parameter is not strong enough to completely entrain the system into the *π* state, on the other hand it is large enough to maintain certain correlations among the instantaneous frequencies of oscillators. As a compromise of this competition, the instantaneous frequencies of oscillators are not locked, but their average frequencies are locked to multiple, discrete constants. Numerically, it is found that, as *p* increases in the regime from 0.69 to 0.71, the fundamental frequency becomes smaller and smaller, i.e., the period becomes larger and larger. In this sense, the *π* state can be regarded as a Bellerophon state with infinite period.

## Discussion

We have performed a full study of synchronization in a Kuramoto model in which both conformists and contrarians coexist. Three specific forms of correlations between the distribution of natural frequencies and the coupling strength of the oscillators have been considered, corresponding to three distinct strategies in which contrarians are progressively flipped to conformists. A detailed and complete analytical treatment of the model has been offered, based on linear stability and mean-field analysis. Several, rigorous, predictions can been made: *i)* the incoherent state is neutrally stable below the synchronization threshold; *ii)* analytical expressions are found for all critical points in the synchronization path; *iii)* all possible stationary coherent states, including the *π* state and two types of travelling wave states, are predicted; *iv)* a novel non-stationary state (the Bellerophon state, in which the oscillators split into multiple coherent clusters) can be fully characterized; *v)* all theoretical predictions are incredibly well verified by means of extensive direct simulations of the model equation, with an amazing accuracy. We emphasize that the above results are obtained by applying a symmetric Cauchy-Lorenz density distribution, which usually is in favor of the analytical treatment for Kuramoto-like models.

When compared with the other coherent states, the Bellerophon state share some similarities with the standing wave state, where two clusters rotate in opposite directions along the unit circle. However, it has two essential differences. First, in the standing wave state, oscillators in each coherent clusters are frequency-locked whereas oscillators in each coherent clusters of the Bellerophon state are not frequency-locked. Second, in the standing wave state, there are only two clusters, while in the Bellerophon state, multiple pairs of clusters of contrarians and conformists coexist, with each pair of clusters rotating (on average) at a different speed.

Furthermore, the Bellerophon state can be compared with the oscillating *π* state^35^. The connections and differences between these two non-stationary states are as follows: *i)* both oscillating *π* and Bellerophon states are characterized by time dependent, quantized clusters in terms of the average frequencies. In the coherent clusters, oscillators are neither phase-locked, nor frequency-locked. However, their average frequencies are locked while their instantaneous frequencies are not; *ii)* the Bellerophon state is a transitional state between the incoherent state and the *π* state, while the oscillating *π* state is a transitional state between the TW state and the *π* state; *iii)* in the oscillating *π* state, the average frequencies of coherent oscillators are locked to even-numbered multiples of a principle frequency, i.e., Ω_*n*_ = ±2*n*Ω_1_ with *n* = 0, 1, 2, …, while in the Bellerophon state, they are locked to odd-numbered multiples of a principle frequency, i.e., Ω_*n*_ = ±(2*n* − 1)Ω_1_ with *n* = 1, 2, …; *iv) n* can take the value 0 in the oscillating *π* state. So the dynamics of oscillators in such clusters is very special, i.e., they must behave like shuttle-run. In fact, it is revealed that they only do shuttle-run in certain limited range of phase space, not the whole range [0, 2*π*]. There is no such counterpart in the Bellerophon state, in which oscillators in all coherent clusters rotate along the whole unit circle; *v)* on the unit circle, in the oscillating *π* state the coherent oscillators form two main clusters which contribute most to the order parameter. These two clusters keep a constant phase difference *π* and do shuttle-run in certain limited range of phase space as a whole. In the Bellerophon state there are four main clusters, i.e., *Cont*(±1) and *Conf*(±1). The motions of these clusters exhibit intermittency. During most of the time, *Cont*1 and *Cont*(−1) are connected just like one cluster, and so do *Conf*1 and *Conf*(−1). In addition, these two clusters keep a phase difference *π*. Then, within a short bursting period, *Cont*1 (*Conf*1) and *Cont*(−1) (*Conf*(−1)) quickly separate and rotate toward opposite directions along the unit circle; *vi)* our numerical results suggest that the Bellerophon state only occurs in *Case* 2, while the oscillating *π* state only occurs in *Case* 3.

As both the oscillating *π* state and the Bellerophon state share essential similarities, they can be actually encompassed under the unified concept of Bellerophon state as a typical non-stationary coherent state in model (2). Our work suggests that, compared with all coherent states studied previously, the Bellerophon states represent a high-order, time-dependent collective behavior in coupled phase oscillators.

Finally, we would like to point out that as *p* approaches 1 in the *π* state regime, the cluster of contrarians gradually shrinks and finally disappears at *p* = 1. In this limit case, the *π* state degenerates into the normal phase-locking coherent state in the original Kuramoto model.

## Methods

### Linear stability analysis

Consider the continuity equation for the density of oscillators, which one can rewrite in the form


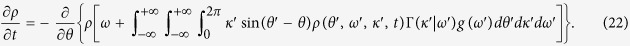


For the incoherent state, *ρ*_0_(*θ*, *ω*, *κ*, *t*) = 1/(2*π*). Now, let a small perturbation from that state


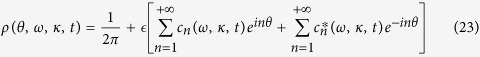


be accounted for, where 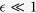, and *c*_*n*_ represents the *n*th Fourier coefficient of *ρ*(*θ*, *ω*, *κ*, *t*). Substituting Eq. (23) into Eq. (22), one gets the linearized characteristic equation


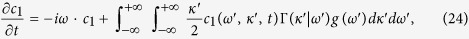


and


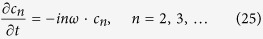


From Eq. (25), it is obvious that the higher Fourier harmonics are neutrally stable to the perturbation^7^,^8^.

Let us now move to derive the characteristic Eq. (10) of the main text for the discrete eigenvalues of the integral operator 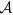, as defined in Eq. (9). With this aim, one seeks for solutions of the first harmonic (hereafter *c* ≡ *c*_1_) having the form *c*(*ω*, *κ*, *t*) = *b*(*ω*, *κ*)*e*^*λt*^, so that the characteristic equation 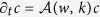 takes the form


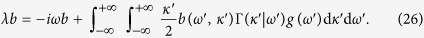


The integral can be evaluated self–consistently by setting 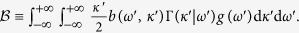


Solving Eq. (26) for the function *b* yields *b*(*ω*, *κ*) = 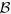/(*λ* + *iω*) for every 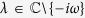. Hence, inserting such an expression for *b* into (26) leads to the characteristic Eq. (10) in the main text, hereafter reported for the sake of clarity:


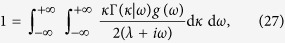


where 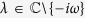. Both real and imaginary parts of Eq. (27) might influence the stability of the incoherent state, so that it is convenient to split Eq. (27) in two conditions, namely


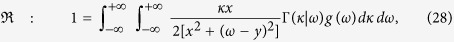



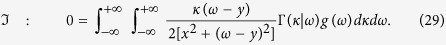


Given Eqs (27, 28, 29), one can predict the critical threshold for the forward PT in model (2). The reasoning to determine the critical threshold for Case 1 has been already discussed, leading to Eq. (12) in the main text. In what follows, therefore we concentrate on the arguments that lead instead to Eqs (14 and 17) in the main text.

Case 2. In this case, substituting the expression (4) for Γ_2_(*κ*|*ω*) into Eq. (28) yields


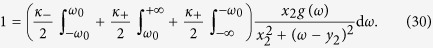


Note that *x*_2_ does not need to be necessary positive in this case. If one takes the critical condition *x*_2_ → 0 for Eq. (30), one obtains


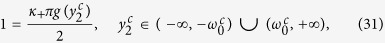


so that the critical fraction of conformists, 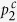, is determined by the Eq. (29), that is


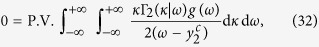


where P.V. stands for the Cauchy principal–value integration within the real line. Notice that 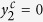 is always a trivial solution of Eq. (32), but it does not satisfy Eq. (31). Nevertheless, there may be more than one value for 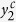 that satisfies Eq. (32). For instance, considering *g*(*ω*) = *g*(−*ω*) and Γ_2_(*ω*, *κ*) = Γ_2_(−*ω*, *κ*), a pair of 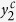 with opposite sign might emerge together, which is indeed the case for a Cauchy–Lorenz FD, as shown by Eq. (15) in the main text.

Case 3. Feeding expression (5) into Eq. (28), and applying the critical condition *x*_3_ → 0, yields


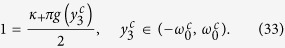


Let us again consider the case of a Cauchy–Lorenz FD. When *κ*_+_ < 2*γ*, a treatment analogous to the one of the previous case leads here to predict that the forward PT does not ever take place, being *x*_3_ always negative (*x*_3_ → 0 is self–contradictory). When instead *κ*_+_ > 2*γ*, then one (from Eq. (29), in the limit *x*_3_ → 0) obtains the equation


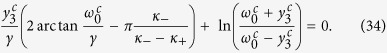


We already discussed in the main text that 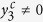, in the regime *κ*_+_ > 2*γ*. Therefore, one must seek for the values of 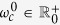 that satisfy Eq. (34). We now prove that no such solutions exist. Let us denote, indeed, with 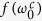 the left–hand side of Eq. (34). Taking the first derivative with respect to 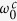 yields


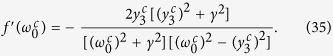


From Eq. (35), it can be seen that 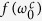 is strictly monotonically decreasing when 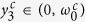, and strictly monotonically increasing when 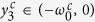. Since 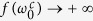 as 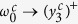, and 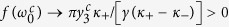 with 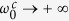, 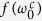 is always positive when 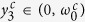. Likewise, 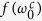 is positive when 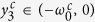. As a result, Eq. (34) has no solution when *κ*_+_ > 2*γ*.

### Mean–field theory

We concentrate here on the details allowing the deduction of eqs (19) and (20) presented in the main text. With this aim, we first rewrite the order parameter (7) in the form


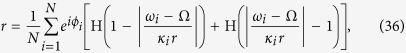


so that locked and drifting contributions are made explicit. For the sake of clarity, its succinct expression 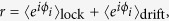 is considered, where the angular brackets denote population averages^8^. Equating real and imaginary parts yields


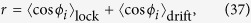



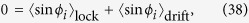


which constitute a closed system of equations for the order parameter of the system.

In the thermodynamical limit, the sums in Eq. (36) are replaced by integrals over the space of probability distributions, so that Eq. (36) can be rewritten in the continuous form


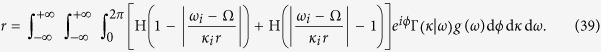


The contributions coming from phase–locked and drifting oscillators can be separately calculated. In the locked state, all those oscillators for which |*ω*_*i*_ − Ω| ≤ |*κ*_*i*_*r*| are entrained (from the self–consistent argument) by the mean–field to the phase 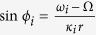. This implies that 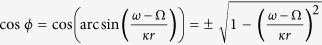, where conformists take the positive sign and contrarians take the negative sign in the first integrand function. This is because (in a stationary coherent state) conformists attempt to follow the global rhythm of the system (and hence cos *ϕ*_*i*_ > 0), whereas contrarians try to oppose the system’s mean–field (implying cos *ϕ*_*i*_ < 0). Therefore, inserting the latter into the locked–contribution to the order–parameter, we obtain


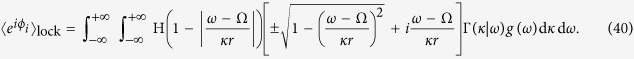


At variance, the drifting oscillators cannot be entrained by the mean-field. In the thermodynamic limit, they are supposed to follow the continuity equation (because of conservation of the number of oscillators). Self-consistently, the drifting oscillators should form (in a stationary state) a stationary distribution on the unit circle^8^. Stationarity requires the probability density *ρ*_*drift*_(*ϕ*|*κ*, *ω*) to be inversely proportional to the velocity field driving the dynamics of the drifting oscillators on the circle, defined by Eq. (8) in the main text. Hence, after appropriate normalization, one finds that the distribution of the drifting oscillators in the rotating frame can be written explicitly as


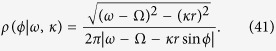


It is easy to observe that (for symmetry reasons) 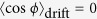, whilst


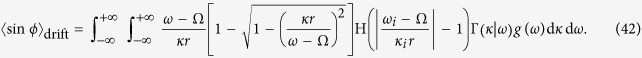


The latter equations tell us that, though the drifting oscillators do not contribute to the real part of the amplitude *r*, they actually contribute to its imaginary part. Therefore, inserting Eqs (40 and 42) into Eq. (39), and equating real and imaginary parts, one finally arrives at the closed system of self–consistent equations given by Eqs (19 and 20) in the main text, that is:


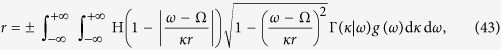



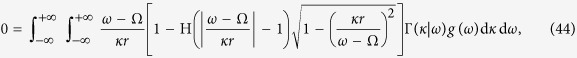


Let us now specify the analysis of the above equations to the three distinct cases considered in the text.

Case 1. Substituting the expression (3) for the distribution Γ_1_(*ω*) into Eqs (43 and 44), yields


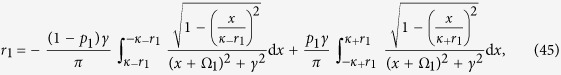



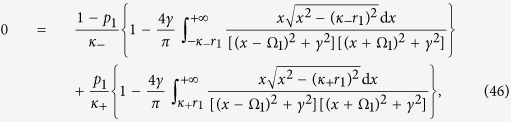


where a Cauchy–Lorenz FD and an appropriate change of variables have been adopted.

It is shown in the main text that (by setting Ω_1_ = 0 and integrating Eq. (45)) one obtains a closed form expression for the order parameter *r*_1_ in terms of the system parameters *γ*, *κ*_−_ and *κ*_+_ given by


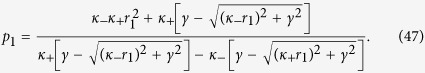


We stress here that, when |*κ*_−_| > |*κ*_+_|, the critical proportion of conformists for the backward phase transition (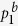) can be determined by setting d*p*_1_/d*r*_1_ = 0 in Eq. (47). Moreover, taking the limit *r*_1_ → 0^+^ in Eq. (47), one recovers the expression (12) for 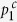. The reasons why 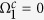 when the incoherent state loses its stability can be understood from two different viewpoints. On the one hand, one can integrate Eq. (45) in the limit *r*_1_ → 0^+^, and obtain


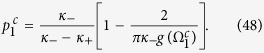


Eq. (48) shows that 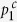 attains its minimum when 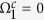, i.e. it corresponds to the foremost critical point for the onset of synchronization. Meanwhile, 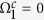 satisfies trivially the phase balance equation, being then the reasonable choice at the critical point. On the other hand, in the linear stability analysis, substituting Eq. (23) into the definition of the complex order parameter *Z* with *c*(*ω*, *κ*, *t*) ∝ *e*^*λt*^, one gets *Z*(*t*) ∝ *e*^−*iyt*^ where *y* is the imaginary part of the complex eigenvalue *λ*. Being Ω the mean–field frequency, one has Ω^*c*^ = −*y*^*c*^, which holds in all three cases. Moreover, it is proved above that 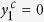 above, therefore the *π* state emerges immediately after the incoherent state loses its stability.

Case 2. Inserting the expression (4) for the distribution Γ_2_(*κ*|*ω*) into Eqs (43 and 44) yields


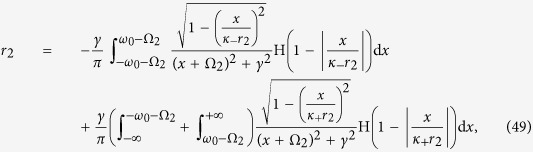



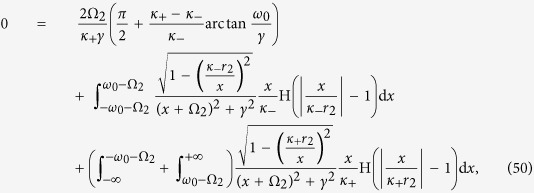


having again adopted a Cauchy–Lorenz FD, and performed an appropriate change of the frequency variables.

Setting Ω_2_ = 0 (which is always a solution of Eq. (50)), Eq. (49) can be integrated with the synchronization condition |*κ*_±_ *r*_2_| > *ω*_0_, giving the following self–consistent equation for *r*_2_:


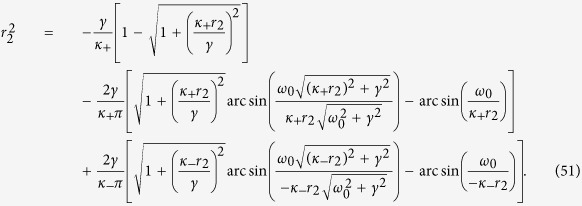


By adopting the same arguments developed in Case 1, one can identify the *π* state, as well as the critical proportion of conformists *p*_2_ where the *π* state loses its stability in the backward phase transition (i.e. 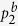). Furthermore, one can also determine the critical proportion 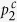 for the forward phase transition. At variance with the previous case, here 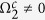, as 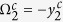, and 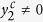 is proved by the linear stability analysis. Moreover, for vanishing 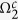 in Eq. (49), one has


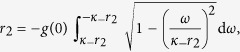


in the limit *r* → 0^+^, which leads to a contradiction. Therefore, in order to determine 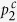, one must seek for solutions of the phase balance Eq. (50). Substituting Γ_2_(*κ*|*ω*) into Eq. (50), and setting the critical condition *r*_2_ → 0^+^, one obtains


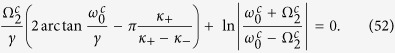


On the other hand, integrating Eq. (49) with the limit *r*_2_ → 0^+^ yields


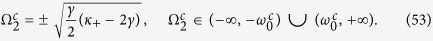


Eqs (52 and 53) are exactly the same as those for the critical point resulting from linear stability analysis, which are fully supported by our numerical simulations (see Figs 1 and 2(d–f) in the main text).

Case 3. In this case, substituting expression (5) for Γ_3_(*κ*|*ω*) into Eqs (19 and 20) yields


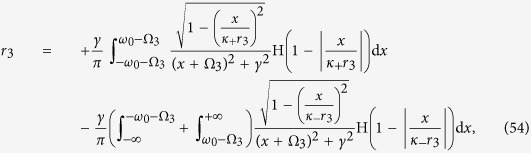



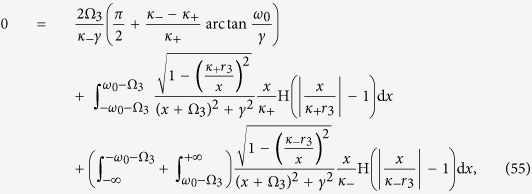


having, once again, chosen a Cauchy–Lorenz FD, and performed an appropriate change of the frequency variables.

Setting Ω_3_ = 0 (which is always a solution of Eq. (55)), and integrating Eq. (54) with the synchronization condition |*κ*_±_ *r*| > *ω*_0_, one can obtain the theoretical characterization of the *π* state by analyzing the self–consistent equation


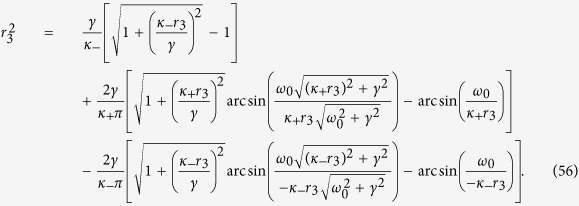


Substituting Γ_3_(*κ*|*ω*) into the balance equation and integrating Eq. (55), one gets


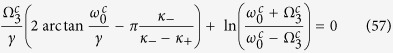


and


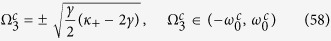


with the critical condition *r*_3_ → 0^+^. Eqs (57 and 58) are consistent with Eqs (33 and 34). Thus, if *κ*_+_ < 2*γ*, the coherent state will never emerge. Otherwise, the incoherent state cannot exist.

## Additional Information

**How to cite this article**: Qiu, T. *et al.* Synchronization and Bellerophon states in conformist and contrarian oscillators. *Sci. Rep.* **6**, 36713; doi: 10.1038/srep36713 (2016).

**Publisher’s note**: Springer Nature remains neutral with regard to jurisdictional claims in published maps and institutional affiliations.

## Supplementary Material

Supplementary video 1

Supplementary video 2

Supplementary Information
