# Supplementary material for: Synchronization and Bellerophon states in conformist and contrarian oscillators
Source: Sci Rep. 2016 Nov 9;6:36713. doi: 10.1038/srep36713 (PMC5101499; doi:10.1038/srep36713)
Supplement: Supplementary Information [file srep36713-s3.pdf]

SUPPLEMENTARY INFORMATION of the Manuscript entitled "Synchronization and Bellerophon states in conformist and contrarian oscillators"

Tian Qiu,[1,2] Stefano Boccaletti,[3,4,*] Ivan Bonamassa,[5] Yong Zou,[1] Jie Zhou,[1] Zonghua Liu,[1] and Shuguang Guan[1,†]

[1]*Department of Physics, East China Normal University, Shanghai, 200241, China*
[2]*Institute of Condensed Matter and Material Physics, School of Physics, Peking University, Beijing, 100871, China*
[3]*CNR–Institute of Complex Systems, Via Madonna del Piano, 10, 50019 Sesto Fiorentino, Florence, Italy*
[4]*The Embassy of Italy in Tel Aviv, 25 Hamered street, 68125 Tel Aviv, Israel*
[5]*Department of Physics, Bar–Ilan University, 592000, Ramat Gan, Israel*
(Dated: September 29, 2016)

Multimedia Material – Characterization of the Bellerophon state.

In Fig. 4 of the main text, the novel emergent states (the Bellerophon states) are characterized by means of *snapshots* of the instantaneous phases θ_i, the average speeds $\langle\dot{\theta}_i\rangle$, and the instantaneous speeds $\dot{\theta}_i$ of the oscillators. One typical Bellerophon state of Fig. 4(b) has been further described in Fig. 5. However, as Bellerophon states are nonstationary, a better visualization of the oscillators' dynamics can be gathered through the monitoring of the temporal evolution of their phases and speeds. To this end, we here enclose two animated movies, which vividly show the collective behavior of the oscillators in the Bellerophon state corresponding to Fig. 4(b). To open them, one can use either Windows Media Player, or DivX Player, but not Quick Time Player. Herewith, we provide a brief description of the two movies:

(movie a) Evolution of the instantaneous phases $\theta_i(t)$ (top), the instantaneous speeds $\dot{\theta}_i(t)$ (middle), and the accumulated average of instantaneous speeds $\langle\dot{\theta}_i(t)\rangle = \frac{1}{t}\int_0^t \dot{\theta}_i(\tau)d\tau$ (bottom) of all oscillators in the state of Fig. 4(b) of the main text. The filmed time window is of the order of one period $T_1 = 1/\Omega_1$.

(movie b) Collective motion of the oscillators forming clusters $Cont(\pm1)$, $Conf(\pm1)$, $Conf(\pm3)$, and $Conf(\pm5)$ (from the inside to the outside, respectively). For a better visualization, we artificially arrange the oscillators forming different clusters in circles of different radii (even though they all, in fact, rotate along the unit circle). As shown in the movie, oscillators in $Cont(\pm1)$ and $Conf(\pm1)$ rotate 1 loop, while $Conf(\pm3)$ and $Conf(\pm5)$ rotate 3, and 5 loops, respectively, within the period $T_1 = 1/\Omega_1$. Oscillators in each cluster generally rotate with heterogeneous speeds. Especially, the motion of oscillators in $Cont(\pm1)$ and $Conf(\pm)1$ turns out to be intermittent. During most of time, $Cont(+1)$ and $Cont(-1)$ merge together as one cluster, and so do $Conf(+1)$ and $Conf(-1)$. During such static periods, the phase difference between them approximately is π, resembling the π state. Actually, as p increases, the period of the motion becomes larger and larger, and finally the system transits into the π state.

*Corresponding author: stefano.boccaletti@gmail.com
†Corresponding author: guanshuguang@hotmail.com
